# The biased nucleotide composition of the HIV genome: a constant factor in a highly variable virus

**DOI:** 10.1186/1742-4690-9-92

**Published:** 2012-11-06

**Authors:** Antoinette C van der Kuyl, Ben Berkhout

**Affiliations:** 1 Laboratory of Experimental Virology, Department of Medical Microbiology, Center for Infection and Immunity Amsterdam (CINIMA), Academic Medical Center of the University of Amsterdam, Meibergdreef 15, Amsterdam, AZ 1105, The Netherlands

## Abstract

Viruses often deviate from their hosts in the nucleotide composition of their genomes. The RNA genome of the lentivirus family of retroviruses, including human immunodeficiency virus (HIV), contains e.g. an above average percentage of adenine (A) nucleotides, while being extremely poor in cytosine (C). Such a deviant base composition has implications for the amino acids that are encoded by the open reading frames (ORFs), both in the requirement of specific tRNA species and in the preference for amino acids encoded by e.g. A-rich codons. Nucleotide composition does obviously affect the secondary and tertiary structure of the RNA genome and its biological functions, but it does also influence phylogenetic analysis of viral genome sequences, and possibly the activity of the integrated DNA provirus. Over time, the nucleotide composition of the HIV-1 genome is exceptionally conserved, varying by less than 1% per base position per isolate within either group M, N, or O during 1983–2009. This extreme stability of the nucleotide composition may possibly be achieved by negative selection, perhaps conserving semi-stable RNA secondary structure as reverse transcription would be significantly affected for a less A-rich genome where secondary structures are expected to be more stable and thus more difficult to unfold.

This review will discuss all aspects of the lentiviral genome composition, both of the RNA and of its derived double-stranded DNA genome, with a focus on HIV-1, the nucleotide composition over time, the effects of artificially humanized codons as well as contributions of immune system pressure on HIV nucleotide bias.

## Review

### Nucleotide composition: the HIV genome

Viruses, whether their genomes are composed of RNA or DNA, and whether they have single (ss)- or double (ds) -stranded genomes, often differ substantially in the base composition of their genomes, compared to each other and to their hosts. Retroviruses are a special class of viruses, as they alternate between a ssRNA (in the virion) and a dsDNA genome (integrated in the host genome). The RNA genome is reverse transcribed into dsDNA by the viral reverse transcriptase (RT) enzyme that generates both strands of DNA using the genomic RNA template for first strand synthesis and the resulting cDNA for second strand synthesis, while the (integrated) dsDNA genome is transcribed into viral RNA by the host RNA polymerase II.

The remarkable adenine (A)-richness of the HIV RNA genome was already noticed several decades ago [1], and was investigated in more detail thereafter [2-5]. Having RNA genomes that are rich in A and low in cytosine (C) was found to be a general property of the lentivirus family [3,5,6] to which HIV belongs, in contrast to e.g. deltaretroviruses such as human T-cell leukaemia virus (HTLV) that possess genomes poor in A and rich in C [2], e.g. 23.6% A, 34.9% C, 18.3% G and 23.2% U for HTLV type 1 (GenBank accession no. D13784).

Although HIV is one of the most variable viruses known with regard to its nucleotide substitution rate, the base composition of the genome is surprisingly stable over time, varying less than 1% per base per isolate whether originating from early or later years of the epidemic and regardless of HIV-1 group or subtype (Table 1). Subtle regional differences in base composition are present across the HIV genome; e.g. the early genes such as *tat*, *rev*, *nef*, and the untranslated 5’ leader RNA are less A-rich than the *pol* gene (Table 2). The stability of the base composition over time also holds when examining smaller genome fragments, e.g. from the *gag* or *pol* genes, and is even true for highly variable genes such as *env*.

**Table 1:** **Average nucleotide composition of full**-**length lentivirus DNA genomes**

**Primate lentivirus**	**No**. **of genomes analysed**	**A(%) ±std**	**C(%) ±std**	**G(%) ±std**	**T(%) ±std**
HIV-1, group M, subtype A	3	35.1 ± 0.2	18.1 ± 0.2	24.4 ± 0.3	22.4 ± 0.4
HIV-1, group M, subtype B	18	35.3 ± 0.2	18.1 ± 0.2	24.4 ± 0.2	22.3 ± 0.1
HIV-1, group M, subtype C	18	35.5 ± 0.2	18.0 ± 0.2	24.2 ± 0.1	22.3 ± 0.1
HIV-1, group M. subtype D	1	35.8	18.0	24.1	22.1
HIV-1, group O	4	35.0 ± 0.2	19.0 ± 0.1	23.8 ± 0.2	22.2 ± 0.2
HIV-1, group N	8 (gag-pol-env only)	36.0 ± 0.2	17.6 ± 0.2	24.0 ± 0.1	22.2 ± 0.2
HIV-1, group P	1	33.9	18.5	24.6	22.7
SIVchimpanzee	7	35.3 ± 0.5	18.3 ± 0.2	23.8 ± 0.3	22.5 ± 0.2
SIVgorilla	4	34.6 ± 0.0	18.5 ± 0.1	24.6 ± 0.1	22.3 ± 0.2
SIVmangabey	3	34.0 ± 0.2	18.9 ± 0.2	25.1 ± 0.3	22.1 ± 0.4
SIVgreen monkey	9	33.6 ± 0.6	19.3 ± 0.7	25.0 ± 0.5	22.0 ± 0.3
SIVmandrill	3	34,6 ± 1.3	18.1 ± 1.8	24.5 ± 0.6	22.9 ± 1.1
HIV-2	16	33.9 ± 0.3	20.4 ± 0.5	24.9 ± 0.3	20.7 ± 0.5
pSIV (lemur endogenous lentivirus)	1^a^	29.0	20.5	27.5	23.1
**Non**-**primate lentivirus**					
EAIV	25	35.7 ± 0.2	16.0 ± 0.3	22.0 ± 0.2	26.4 ± 0.4
CAEV/Ovine lentivirus^b^	7/4	38.0 ± 0.5	15.7 ± 0.6	25.2 ± 0.4	21.1 ± 0.4
CAEV subtype E	2	33.9 ± 0.3	28.1 ± 0.0	28.1 ± 0.2	19.5 ± 0.0
Maedi-visna virus	6	37.2 ± 0.2	26.0 ± 0.1	26.0 ± 0.1	21.4 ± 0.0
Jembrana disease virus	1	31.7	20.1	26.4	21.9
BIV	1	31.8	21.2	23.8	23.2
FIV cat/cougar	4/14	38.0 ± 0.3	14.9 ± 0.1	22.0 ± 0.3	25.2 ± 0.3
FIV Pallas’ cat/lion^c^	1/2	38.0 ± 0.1	13.7 ± 0.3	22.1 ± 0.3	26.2 ± 0.6
RELIK (hare endogenous lentivirus)^d^	1 (gag-pol-env only)	34.0	19.4	22.4	24.1
ELVmpf (ferret endogenous lentivirus)	1	33.6	20.0	23.6	22.8

^a^ Consensus sequence of pSIVgml (gray mouse lemur, one proviral copy) and pSIVfdl (fat-tail mouse lemur, several proviral copies).

^b^ Viruses labeled CAEV subtype E and Maedi-visna virus, respectively, different significantly in nucleotide composition from other CAEV types including viruses labeled ovine lentivirus.

^c^ FIV isolated from cats and cougars is phylogenetically distinct from FIV found in Pallas’ cat and lions.

^d^ Similar frequencies for rabbit endogenous retrovirus sequences.

Std = standard deviation.

**Table 2:** **HIV**-**1 nucleotide composition of genome segments**

**HIV**-**1 genome segment**^**a**^	**Length(nt)**	**A (%)**	**C (%)**	**G (%)**	**U (%)**
LTR (R3-U-R5)	635	25.0	24.4	27.2	23.3
Gag-ORF	1503	36.9	19.6	24.5	19.1
Pol-ORF	3012	38.9	16.5	22.8	21.9
Env-ORF	2571	34.7	17.1	24.0	24.3
Vif-ORF	579	36.1	18.0	24.0	21.9
Vpr-ORF	292	32.5	18.5	26.7	22.3
Tat-ORF	306	33.0	23.9	24.2	19.0
Rev-ORF	351	29.9	23.1	28.2	18.8
Vpu-ORF	249	38.6	11.7	26.5	23.3
Nef-ORF	621	30.6	21.3	28.2	20.0

^a^HXB2 reference strain (GenBank acc. no. K03455).

The intriguing question is then: how can such a variable virus maintain such stable nucleotide frequencies? For this, we have to consider the various aspects of the HIV replication cycle in the host cell that influence the RNA and/or DNA genome. Reverse transcription, translation, splicing, encapsidation in virions to name a few, all have their own requirements with respect to the viral RNA genome. And how are viral genomes with a deviant nucleotide composition selected against, or selected for? A recent paper suggests that RNA genome structure, and not the encoded proteins, is the most decisive factor that triggers HIV-1 conservation [7]. In this review, an overview of lentiviral genome composition characteristics will be given with an emphasis on HIV-1, elucidating possible mechanisms for the generation of this bias and the biological consequences.

### A-bias of HIV and other lentiviruses

The RNA genomes of HIV-1 group M virus isolates contain a similar amount of A-nucleotides as those of group O (35%, Table 1). Group N and P viruses appear to contain slightly higher (group N) or lower (group P) levels of A-nucleotides, but only one (group P) or no (group N) full-length genomes with long terminal repeats (LTRs) are available for these groups (Table 1). As the LTR is relatively A-poor [5], calculations based upon the coding regions only will result in higher A-levels. Interestingly, HIV-1 group M subtypes A, B, C and D have significantly different nucleotide compositions concerning the A- and G-percentages (for 6 out of 6 comparisons p<0.05), but less so for the C- and U-nucleotide levels (only for 3 out of 6 comparisons p<0.05) (Table 3). This suggests that HIV-1 group M subtypes have dissimilar nucleotide compositions; the A and G levels are variable, while the C and U levels are more conserved. The time period elapsed since the subtypes shared a common ancestor could account for these differences. The recombinant CRF02_AG strain did not differ appreciably in genome composition from its subtype A parental strain with whom it shares the larger part of its genome, but it was significantly divergent from the other parent that belongs to subtype G (Table 3).

**Table 3:** **Nucleotide composition of the different HIV**-**1 subtypes** (**group M**)

**Comparison**	**P**-**value**^**a**^**for A**-**content difference**	**P**-**value for C**-**content difference**	**P**-**value for G**-**content difference**	**P**-**value for U**-**content difference**
**Subtype B**/**subtype A**^**b**^	0.0008	0.003	0.01	0.03
**Subtype B**/**subtype C**	0.0002	<0.0001	<0.0001	0.0004
**Subtype A**/**subtype C**	0.03	0.03	0.007	0.12
**Subtype B**/**subtype D**	<0.0001	0.70	<0.0001	0.29
**Subtype A**/**subtype D**	<0.0001	0.20	<0.0001	0.22
**Subtype C**/**subtype D**	<0.0001	0.02	<0.0001	0.009
**Subtype A**/**subtype G**	<0.0001	0.88	<0.0001	0.04
**Subtype A**/**CRF02**_**AG**	0.84	0.25	1.00	0.30
**Subtype G**/**CRF02**_**AG**	<0.0001	0.45	<0.0001	0.004

^**a**^ Differences in nucleotide composition between HIV-1 subtypes were analysed using Student’s t-test.

^**b**^ Based on 41 subtype B, 62 subtype A, 55 subtype C, 38 subtype D, 18 subtype G and 32 recombinant CRF02_AG gag-pol-env sequences.

Among the simian immunodeficiency viruses (SIV), isolates from chimpanzees have the highest A-content (35.3%), comparable to the HIV-1 viruses. HIV-1 group M, N, and O viruses are all likely descendants from independent cross-species transmissions of SIVcpz, although it is debatable whether group O viruses were transmitted from chimpanzees to gorillas and then to humans, or directly to humans from chimpanzees, as group O viruses fall within the SIVcpz cluster, but similar strains have been detected in gorillas only and not in chimpanzees (for a review see [8]). Another SIV from gorillas, SIVgor, is the probable origin of HIV-1 group P [9]. SIVgor has indeed a lower level of A-nucleotides, similar to the group P virus. SIV from other monkeys, including HIV-2 that originates from mangabeys, also contain somewhat lower A-levels ranging than SIVcpz or HIV-1 (Table 1).

Bovine immunodeficiency virus (BIV) and the related Jembrana disease virus have the lowest percentage of A-nucleotides (31.7%) of all exogenous lentiviruses analysed.

Interestingly, the endogenous lentiviral genomes detected in prosimians [10,11], and estimated to be between 4 and 14 million years old, have an even lower A-count (29.0%), although A remains the most frequently used nucleotide. In contrast, endogenous lentiviral sequences from rabbit [12,13], hare [14], and ferret [15], all estimated to be at least 7, but more likely at least 12 million years old, have A-counts more similar to that of exogenous lentiviruses (around 34%). In line with the nucleotide characteristic of exogenous lentiviruses, the endogenous lentiviruses are also C-poor (Table 1).

Feline immunodeficiency virus (FIV) strains and some viruses belonging to the caprine/ovine lentivirus group display the highest A-nucleotide percentage of all lentiviruses (maximum of 38.0%), with a concomitant drop in C-count to minimally 13.7%. Among the ovine/caprine lentiviruses, the nucleotide composition differs significantly between subgroups. Caprine arthritis-encephalitis virus (CAEV) subtype E isolates have A-nucleotide levels as low as 33.9% , while viruses labelled maedi-visna virus have increased A-levels reaching 37.2%. CAEV non-E subtypes together with viruses classified as ovine lentiviruses display frequencies of A-nucleotides up to 38.0%.

Among lentivirus groups for which more than a single isolate could be analysed, the percentage of each nucleotide is stable, and the overall nucleotide composition serves as a distinguishing trait. For instance, HIV-2 is the only lentivirus that has similar levels of C and U nucleotides (20.4% and 20.7%, respectively), and this characteristic is found in all full-length HIV-2 genomes available, except for one that was described as a highly divergent strain of HIV-2, probably originating from another zoonotic transmission [16].>

### Nucleotide composition of HIV-1 over time

The nucleotide composition of HIV-1 has remained remarkably constant over the three decades that virus variants from the current epidemic have been monitored (Table 4). Early isolates (1983–1997) of HIV-1 group M (analysed for subtypes A and B), N, and O viruses do not significantly differ from more recent isolates (1998–2009) with regard to nucleotide composition (Table 4). Comparing isolates obtained before 1990 to isolates obtained after the year 2000, or other variations of the time window, did not change the results (not shown). This suggests that the precise nucleotide composition of the HIV-1 genome, and most likely of other lentivirus genomes is a stable and unique trait that is highly preserved throughout evolution.

**Table 4:** **Nucleotide composition of the HIV**-**1 RNA genome over time** (**1983**–**2009**)

**HIV**-**1 group**/**subtype**	**Number of genomes analysed**^**a**^	**Average nucleotide composition % 1983-1997**^**b**^	**Range**	**Number of full-length genomes analysed**	**Average nucleotide composition % 1998**-**2009**^**b**^	**Range**
**M**, **subtype A**^c^	24	A 36.5	35.8-36.9	38	A 36.6	36.3-36.9
		C 17.5	17.3-17.8		C 17.5	17.0-17.7
		G 23.8	23.3-24.4		G 23.8	23.4-24.2
		U 22.3	21.8-22.6		U 22.1	21.8-22.5
**M**, **subtype B**^d^	16	A 36.7	36.5-37.0	25	A 36.7	36.3-37.0
		C 17.4	17.1-17.7		C 17.4	17.1-17.7
		G 23.7	23.5-24.0		G 23.7	23.4-24.1
		U 22.2	22.0-22.4		U 22.3	22.1-22.5
**N**^e^	2	A 35.7	35.6-35.8	6	A 36.1	36.0-36.4
		C 17.7	17.6-17.9		C 17.5	17.2-17.9
		G 24.2	24.1-24.3		G 23.9	23.9-24.1
		U 22.3	22.2-22.3		U 22.2	22.0-22,4
**O**^d^	4	A 35.4	35.0-35.8	3	A 35.3	34.8-36.1
		C 18.7	18.6-18.8		C 18.8	18.3-19.1
		G 23.7	23.5-24.2		G 23.8	23.3-24.1
		U 22.2	21.9-22.4		U 22.1	21.9-22.3
**P**^f^	0	NA	NA	2	A 34.1	33.9-34.4
					C 18.4	18.3-18.5
					G 24.5	24.4-24.6
					U 22.4	22.2-22.6

^a^ Gag-pol-env only.

^b^ No significant differences in nucleotide composition were scored between groups over time (p>0.05, Student’s t-test).

^c^ A shorter sequence of HIV-1 subtype A was analysed (approx. 8600 nt), as not many full-length genomes were available.

^d^ Only genomes with ≤ 10 ambiguous nucleotides were used for the analysis.

^e^ Seven of eighth group N genomes contain ambiguous nucleotides (range 5–38).

^f^ Only two full-length genomes of group P viruses are available from the Los Alamos Database, which contain 54 and 66 ambiguous nucleotides, respectively.

Unfortunately, of the early HIV-1 group M viruses from 1959 and 1960, only very short fragments (< 200 nucleotides) have been amplified, from which the genomic nucleotide composition cannot be reliably estimated [17,18]. For HIV-1 group O, a total of 1770 nucleotides of four *pol* gene fragments are available for an isolate recovered from autopsy material collected in 1976 of a father and daughter infected in the 1960s [19]. The base composition of these combined fragments is 39.7% A, 15.6% C, 22.4% G and 21.8% U, which, except for G, falls within the range of the base composition of homologous *pol* gene fragments from group O isolates from 1986–1995 (ANT70 from 1986 acc. no. L20587; MVP5180 from 1991 acc. no. L20571; pCMO2.3 from 1995 acc. no. AY618998): 39.2-40.3% A, 14.7-15.7% C, 22.7-23.6% G and 21.9-22.3% U.

### HIV nucleotide composition and drug-resistance

Antiretroviral therapy (ART) targeting HIV has been available from the 1990s, with effective treatment involving multiple drugs being instituted since 1995. Most antiretroviral drugs target the products of the *pol* gene, especially the protease and RT. HIV rapidly develops drug resistance when only one or two drugs are given, or when compliance is low. The dominant type of mutation involved in the generation of drug-resistance is G-to-A [20], which on a microscale could further increase the A-content. To test the hypothesis that ART use could change HIV-1 genome composition, we have compared the sequence composition of approximately 1300 nt of the *pol* gene of blood plasma virus of 38 untreated Dutch patients MSM (men having sex with men) infected with HIV-1 subtype B, with *pol* gene sequences of 14 ART treated Dutch patients (MSM) infected with subtype B virus, five of which failed therapy due to the emergence of drug resistant virus. Comparison of the nucleotide composition of the *pol* gene between the two groups showed no significant statistical difference (e.g. p = 0.13 when comparing the % of A-nucleotides in both groups), and there was also no significant difference between *pol* genes with or without drug resistance mutations (p = 0.76). This suggests that replication in the presence of ART, which frequently includes nucleotide analogue drugs, and the selection of drug-resistance mutations do not appreciably change the nucleotide composition of the HIV-1 *pol* gene and most likely, of the complete genome.

### Nucleotide composition and RNA structure

Inside the HIV-1 virus particle, two copies of its single-stranded RNA genome are packaged, together with virus-encoded proteins and cellular RNA molecules including tRNA^lys3^ that acts as a primer for reverse transcription [21,22]. Packaging signals in the viral RNA are essential for its incorporation into an assembling virion particle [23], as are signals for RNA dimerization and nucleocapsid (NC) protein binding [24]. The NC protein covers the complete viral RNA genome and could thus be a sensor of nucleotide composition (for a review, see [25]). This mechanism could underlie the differences in genome composition described for the HIV-1 groups and subtypes, and for HIV-2. Most likely RNA structure and not the primary sequence, is a determining factor here; but the formation and stability of such RNA structures are influenced by the base composition of the HIV-1 genome. This seems particularly true because the biased nucleotide composition is distorted even further when the structured and unstructured regions of the HIV-1 RNA genome [26] were analysed separately. The percentage of A-nucleotides is particularly low in double-stranded structures (21%) compared to the single-stranded parts (79%) (van Hemert et al., submitted for publication). In contrast, the majority of C-nucleotides are present in structured regions (62%) over single-stranded parts (38%).

The length of the RNA molecule plays a role in efficient packaging; HIV-1 based lentiviral vectors do tolerate genomes up to 18 kilobase in size, but infectious titers decline with insert size, probably due to encapsidation difficulties [27]. Genes or gene fragments from multiple sources (e.g. bacterial, viral, and human) are tolerated by these HIV-based vectors, suggesting that the nucleotide composition of the inserted fragment is not critical for packaging.

### Dinucleotide composition of the HIV-1 genome

Not surprisingly given the A-richness of HIV genomes, AA is the most common dinucleotide in HIV-1: 12.5% of dinucleotides in *gag*, 13.7% in *env*[28], while CG is found at the lowest frequency: 1.0% in *gag*, 0.6% in *env*[2,3,28]. Rima and McFerran [29] showed that CpG is actively suppressed in viral genomes and that the low numbers are not the result of a low genomic C+G content, in other words, CpG is suppressed but not GpC. The actual number of CpG dinucleotides was always much lower than expected based on the C+G content of diverse viruses. According to a statistical codon-based model developed by Pedersen *et al*. the nucleotide bias in HIV-1 could be totally explained by negative selection on CpG dinucleotides [30]. However, such a model only partly explains the observed bias, as it does not clarify the preference for A-nucleotides. Active selection against CpG dinucleotides was postulated as an explanation for the low CG content in the lentivirus family. Negative selection against CpG methylation of the DNA genome or a biased mutational spectrum of the reverse transcriptase was proposed as sources of the low CG content of onco-retroviruses [31]. However, the human genome in general also has a lower than expected number of CpG dinucleotides, and the mutation rate of this motif is at least tenfold higher than other dinucleotides, especially in higher primates [32]. This is probably due to cytosine methylation of CpG motifs which results in deamination to thymine [32].

It has been speculated that a low CpG dinucleotide content in lentiviruses protects against methylation of this motif and inactivation of the integrated provirus [2,31]. The newly made and unintegrated HIV DNA is not methylated [31]. Transcriptionally latent HIV-1 proviruses are methylated at two CpG motifs near the transcription start site (reviewed in [33]), suggesting that even low numbers of CpG dinucleotides suffice for methylation and subsequent transcriptional repression. Possibly, low amounts of CpG motifs represent a balance between transcriptional repression and activation from latency that is beneficial for the virus, as HIV-1 could have evolved to avoid CpG dinucleotides in the promoter region. Analysis of other virus families showed that most small virus genomes (<30kb), whether DNA or RNA, are CpG suppressed, a phenomenon not observed in large viruses (>30kb), except for gammaherpesviruses [34]. Only members of the Togaviridae, a virus family with plus-strand ssRNA genomes that have a relatively high genomic CG content (~ 50% in the alphavirus group; ~70% in rubella viruses), are not CpG suppressed [34,35]. Thus, CpG suppression is also present in many RNA viruses that lack a DNA phase in their replication cycle, and are thus not subjected to DNA methylation [29,34]. This observation suggests that evasion of DNA methylation cannot be the sole reason for the low CpG content of most viral genomes. Codon usage or amino acid preferences were ruled out as the driving factors in CpG depletion during virus evolution [29,34]. Other explanations may be needed to explain the low CpG levels [34], such as recognition of nucleotide signature by components of the innate immune system. Factors of the innate immune system and the relation with genomic nucleotide composition or sequences are discussed below (see: Innate immunity and nucleotide composition).

### Nucleotide composition: the viral genes and regulatory elements

The main open reading frames of HIV-1 are *gag* (encoding the matrix (p17^MA^), capsid (p24^CA^), nucleocapsid (p7^NC^), p1^gag^, p2 ^gag^ and p6 ^gag^ proteins), *pol* (encoding the protease, reverse transcriptase, and the integrase enzymes), and *env* (encoding the envelope proteins gp120 and gp41). Small, overlapping open reading frames encode additional proteins such as Vif, Vpu, Vpr, Nef, Tat, and Rev. Although lentiviral genomes are uniformly rich in A-nucleotides, there is local variation. Nucleotide percentages for the individual HIV-1 genes are shown in Table 2. For instance, the *pol* gene of HIV-1 is more A-rich than *gag*, which in turn is more A-rich than *env*[1,3,5]. Overlapping HIV-1 reading frames that encode Tat, Rev and Nef, the latter overlapping with the LTR, contain a lower percentage of A nucleotides than the *gagpol* region [3,5], possibly because the double coding capacity limits the number of A nucleotides that can be accommodated. The LTR promoter region may contain less A nucleotides to expose important regulatory DNA/RNA elements that are A-rich, such as the TATAA box for transcription initiation and the AATAAA motif for transcription termination [36,37]. In general, early genes such as *tat* are less A-rich than late genes [38]. It is possible that the typical nucleotide composition is used to restrict the stability of local RNA structures [39]. In particular, A-nucleotides have limited base pairing potential (only with U, and A-U is a relatively weak base-pair). Clustering A-nucleotides in the ss-genomic regions would prevent the formation of inhibitory RNA structures. To investigate this hypothesis, Keating *et al*. modified the HIV-1 *gag* and/or *pol* genes so that up to 4% of the A-nucleotides were mutated without altering the encoded amino acids or affecting known regulatory sequences [39], including the AU-rich instability elements important for Rev function [40]. Reducing the A-content of the *gagpol* ORF from 36% to 32% resulted in increased RNA stability but also a reduction in cDNA synthesis, suggesting that excessively stable RNA structures can interfere with reverse transcription [39]. In cell culture, reduced viral replication in peripheral blood mononuclear cells (PBMCs) was scored for viruses with modified *gag*-codons, but viral protein expression and viral entry were unaffected. Viral constructs that were codon-modified in *pol* or *gagpol* were infectious, but did barely replicate in PBMCs. These constructs produced strongly reduced levels of infectious particles upon DNA transfection of 293T cells, and the stability of the dimeric RNA genome that is packaged in virions was significantly increased compared to unmodified HIV-1 [39]. Modifying the A-content of *gag* seems to have less effect on viral replication than modification of the *pol* gene, suggesting that local variation in A-richness may reflect functional differences in sequence requirement.

### Codon usage in the HIV-1 genome: triplet analysis, protein composition and translation

Codon bias is the tendency of an organism to encode amino acids by a non-random usage of the 61 degenerate codons available to specify the 20 amino acids during protein synthesis (for a review, see [41]). Codon bias has been described for many organisms, and is usually linked to specific tRNA levels that are mainly determined by the number of tRNA genes that encode a certain tRNA [42]. In humans, tissue specific differences in tRNA expression levels have also been documented [43]. Codons for rare human tRNAs are often found clustered in only a subset of genes, and have been associated with expression modulation of mRNA translation [44,45]. Both highly and lowly expressed human genes can contain such rare codon clusters, and it has been speculated that the proteins encoded by the corresponding mRNAs are not co-translationally folded [44]. It has also been suggested that the use of codons with rare anti-codon tRNAs in an mRNA increases the translational accuracy [45].

The remarkable nucleotide composition of the HIV genome results in a codon bias that is quite different from that of the human genome [1,3,4,46-49]. In summary, given a choice, HIV-1 almost always prefers the most A-rich codon to encode a certain amino acid. Especially the more flexible third codon positions are preferentially occupied by A-nucleotides, e.g. in the *pol* gene the A-content goes up from 34.3% in the first codon position to 46.5% in third positions [4]. In HIV-1 genomes, amino acids that are encoded by A-rich codons are preferentially selected. For example, HIV encoded proteins are relatively rich in lysine (codon = AAR) and poor in proline (codon = CCN), while the opposite is true for similar proteins encoded by the C-rich HTLV retrovirus [3,4,50]. A similar amino acid composition is seen in proteins encoded by other A-rich pathogens such as influenza virus, and in several bacterial proteins, but not in vertebrate ones [50]. Codon usage in HTLV is also different from the human codon use, even with a preference for C-rich codons [5]. Possibly, the use of rare codons in retroviral genes is associated with protein processing, as suggested for human genes [44].

Protein characteristics are affected by biased codon use, as the prevalence of hydrophobic and amphypathic amino acids increases at the expense of hydrophilic residues with increasing G/C-use in the third codon position, which influences protein hydropathy and stability as hydrophobic proteins are generally more stable [51]. In eukaryotic proteins, decreasing hydrophobicity values are accompanied by an increase in the percentage of cysteine residues [51]. Cysteines can form disulfide bridges that enhance protein stability. For instance, HIV Env-gp120 protein contains nine disulfide bridges [52], possibly related to the low number of hydrophobic amino acids as dictated by the low GC-content of the viral codons. Thus, biased codon use may lead to the expression of viral proteins that have an appreciably different composition and characteristics than host cell proteins.

It has been speculated that the HIV-1 codon bias leads to suboptimal protein expression in infected cells due to the limited availability of the matching tRNAs. To express large quantities of HIV-1 proteins for vaccine purposes, codon-optimized viral genes were constructed that are better adapted to the tRNA pool of the host cell [53-55]. Indeed, protein expression increased significantly, although not necessarily due to enhanced translational efficiency, because the mutational inactivation of instability elements resulted in increased mRNA stability [54,55]. Also, altered export of nuclear RNA contributed significantly to increased protein production levels [56]. The requirement of Rev to export the unspliced and singly-spliced HIV-1 mRNAs from the nucleus was lost, probably due to inactivation of the AU-rich instability elements [56].

If HIV-1 gene expression can so easily be upregulated by adapting codons to better suit the hosts tRNA population, why does HIV-1 not change its strategy so that it can produce more offspring? First of all, there is good evidence that HIV-1 gene expression is not maximal, but fine-tuned to allow regulation of diverse processes such as transcriptional activation by the Tat protein and the nuclear export of unspliced HIV-1 transcripts by the Rev protein, and possibly to avoid cell toxicity [57]. Second, HIV-1 expression levels are not at all that low in HIV-1 infected cells, as one out of every 143 cellular transcripts is of viral origin 24 hours after infection as determined by SAGE analysis and high-throughput sequencing [58]. Human endogenous retrovirus (HERV) transcripts were expressed in that study at a ratio of 1:237, highly expressed human genes were found at a ratio of approximately 1:10, but on average human genes were expressed at a ratio of 1:2 million transcripts [58]. It is also important to realize that the codon-optimized genes have been tested in uninfected cells, which may have a different tRNA profile than HIV-1 infected cells. A first indication for this idea comes from the work of van Weringh *et al*. [38], who reported differences in the tRNA pool of HIV-1 infected versus uninfected cells. Analysing the tRNA species present in HIV-1 particles, many tRNAs besides the reverse transcription primer tRNA^lys^ were found [38]. In fact, these tRNAs match well with the typical HIV-1 codon use. If tRNA packaging in the virion would occur without specificity, except for the tRNA^lys^ primer, the tRNA pool of the virion may reflect that of the cell at the time of virion production. Therefore, the authors speculate that at later stages of infection the tRNA pool has changed to suit the translation of HIV-1 late proteins, possibly because the normal tRNA pool has been exhausted by translation of the HIV-1 early proteins. So, in line with the observation that HIV-1 replication and protein expression occur at high levels in infected human cells, the codon bias may reflect the altered milieu of the virus-infected cell.

In addition, there is some evidence that HIV-1 is adapting its codon use to better suit the host’s tRNA pool; e.g. Meintjes and Rodrigo [59] analysed partial *env* gene sequences from 8 HIV-1 infected patients over time (from seroconversion till the AIDS phase) and found that codon use in later *env* genes was more similar to that of the host than that in early samples. During 23 years of the epidemic (1983–2005), an analysis by Pandit and Sinha in 2011 also suggested that codon use in *env*, but more pronounced in *rev* and *tat*, was becoming more similar to that of the human genome, although their study also implicated an unexpected reversal of the effect in later years [60]. Interestingly, no adaptation to chimpanzee codon use was found for SIVcpz despite its relatively long evolutionary time spent with its host [60].

### Effect of the viral Reverse Transcriptase and lack of dUTPase

Multiple enzymes are involved in replication of the HIV-1 genome, each with their own unique error rate. Replication of proviral DNA by host DNA polymerases and transcription of the proviral genome by the host RNA polymerase II is probably not that error prone, as these processes are subject to proofreading [61,62]. The viral Reverse Transcriptase (RT), an RNA/DNA-dependent DNA polymerase, lacks 3’-5’ exonuclease proofreading activity [63], and has therefore been implicated in the high mutability of HIV-1 (see [63,64]). Indeed, when copying LTR sequences, HIV RT was more mutagenic than RNA polymerase II, suggesting an important role of the former in creating viral variability [65]. A recent study suggests that the mutation rate of HIV RT might be lower than early estimates, but it is still significant at 1.4 x 10^-5^ mutations/bp/cycle [66]. Error rates of HIV-1 RT differ with respect to the nucleic acid template, e.g. the enzyme is more accurate while copying RNA than DNA [67]. The fidelity of HIV RT is different for distinct nucleotides, so that the creation of mismatches is not completely random, e.g. misincorporation of dATP is negligible, low for dGTP, but substantial for dCTP and dTTP [64]. This could imply that HIV-1 RT does not actively contribute to the A-nucleotide content of the viral genome. However, at low dCTP or dATP concentrations, HIV-1 RT induces G-to-A and U-to-C hypermutation of the viral DNA, respectively [68]. In blood T lymphocytes, the natural host cell for HIV-1, dCTP levels are much lower than dATP levels [69], which could drive G-to-A hypermutation in HIV-1. The frequent G-to-A mutations observed in the HIV-1 genome that were initially attributed to RT can however also be assigned to the action of host enzymes of the APOBEC3 (A3) family [20,70-72], which will be discussed below.

Another factor relevant for genome modification is the absence of dUTP pyrophophatase (dUTPase) activity encoded by HIV-1. The nonprimate lentiviruses FIV, CAEV, equine infectious anemia virus (EIAV), and the betaretroviruses all encode such an enzyme in the *pol* gene [73]. This enzyme reduces dUTP levels in the cell, such that incorporation of dUTP into the nascent DNA is minimized during cDNA synthesis. Misincorporation of dUTP for dCTP during cDNA synthesis results in G-U mismatches that eventually result in GC→AT transitions. Such a dUPTase gene is absent from all exogenous primate lentiviruses, although a similar sequence was once described in the HIV-1 *envgp120* open reading frame [74]. Deletion or disruption of dUTPase gene in CAEV [75] and FIV [76] induced G-to-A transitions in the viral genome, in line with the frequent incorporation of dUTP opposite G during first-strand cDNA synthesis. However, as HIV-1 normally replicates without a viral dUTPase it may have found alternative ways to circumvent excessive dUTP incorporation [77]. HIV-1 RT was found to efficiently discriminate between dUTP and dTTP *in vitro*, suggesting that HIV-1 DNA synthesis is not affected by the presence of dUTP [78]. However, G-to-A is the premier type of mutation scored during HIV-1 evolution [79,80], which likely also relates to the absence of dUTPase activity.

### Innate immunity and nucleotide composition

Proteins of the innate immunity system recognize the sequence or structure of invading viral RNA or DNA molecules. The overall nucleotide composition as well as specific sequence motifs, such as dinucleotides, are important determinants in the recognition by and escape from these sensors. It has been suggested that the biased nucleotide composition of HIV-1 is directly responsible for the induction of the type I interferon response, as “humanized” *gag*, *pol* and *env* RNA transcripts that were codon-optimized to resemble human genes, lost the ability to induce IFN-α/β production *in vitro*[81]. *A priori*, it seems more likely that particular sequence elements or certain HIV-1 RNA structures trigger an innate immune response than the overall base composition of the HIV-1 genome.

APOBEC proteins are cytidine deaminases involved in innate immunity that target retroviruses (for a recent review, see [82]). These enzymes act on single-stranded DNA generated during reverse transcription to catalyze deamination of dCTP to dUTP. The sequence context is important, targeting CC (APOBEC3G, underlined C is deaminated) or TC (other A3 proteins) in the HIV-1 minus-strand genome [83], which translates to G-to-A mutations in the plus-strand genome, in a similar fashion as dUTP incorporation. HIV-1 genomes carry relatively high numbers of (complementary) GG and GA dinucleotides in the plus-strand [28]; probably because the viral Vif protein counteracts APOBEC3G and 3F, thus relieving APOBEC pressure on the virus (see [84]). If unhindered, APOBEC3G or 3F action would result in G-to-A mutations in the viral plus-strand, and could thus increase the percentage of A-nucleotides in the HIV-1 genome, providing that no excessive hypermutation occurs, which would render the genome non-infectious [85]. However, recent research suggests that even a single “APOBEC-unit” of an infectious HIV-1 particle will edit the virus genome extensively, making APOBEC hypermutation an “all or nothing” phenomenon [86]. A gradient in APOBEC3 editing along the genome has been observed that reflects the viral replication strategy [87]. This would imply that low-level APOBEC mutations are not likely to occur and thus do not contribute to the evolution and the A-richness of the HIV-1 genome. As it stands the frequent G-to-A mutations observed in HIV-1 could still be attributed to the RT enzyme operating at low dCTP levels in virus infected cells [68], as was also predicted by a computer model analysing the bias in HIV-1 *pol* nucleotide misincorporation [88], or possibly to dUTP incorporation during reverse transcription.

Not all proteins of the innate immunity system modify viral nucleic acids. Many are specialized in recognition of certain motifs, dinucleotides or more extended signatures to induce the corresponding signaling pathway to trigger cytokine secretion and immune activation. Avoiding these motifs might thus confer an advantage to the virus, and escape from these factors might influence viral genome composition. Unmethylated CpG motif-containing bacterial and viral DNA is recognized by toll-like receptor 9 (TLR9), a pathogen sensor of the innate immune system that is localized on the endoplasmic reticulum and highly expressed by plasmacytoid dendritic cells (pDCs) [89]. However, as HIV-1 probably does not replicate in pDCs [90], except for thymic pDCs [91], it is not likely that cytosolic DNA would be available for TLR9 stimulation. The optimal DNA recognition motif for human TLR9 is GTCGTT [92], which is on average present only once per HIV-1 genome [93] and absent from the HIV-1 reference strain HXB2, making it uncertain whether HIV-1 DNA is targeted by TLR9. In HIV-1 infected individuals, responses to TLR9, but not TLR7 or TLR8 stimulation were universally decreased compared to uninfected controls [94], possibly due to Env-mediated suppression of TLR9 function [95]. The active suppression of TLR9 by a viral protein, combined with the near absence of binding motifs, may suggest that TLR9 was once involved in an anti-SIV response in primates, but that the virus has successfully circumvented this restriction during evolution. Toll-like receptors 7 and 8 (TLR7 and TLR8) are closely related receptors in endosomes of dendritic cells and macrophages that recognize guanosine- and uridine-rich (GU-rich) stretches in foreign long ssRNA molecules such as uridine-rich stretches in the HIV-1 LTR [96] and other genomic regions [97]. HIV-1 viral RNA was found to induce interferon-alpha secretion by pDCs through TLR7 or TLR8 stimulation [96,98]. This recognition leads to strong immune activation, and HIV-1 has apparently not been able to circumvent this activity [96-99]. In the HIV-1 reference strain HXB2, at least 9 uridine-rich 20-mers have been identified in the ORFs that stimulate TLR7/8 [97], suggesting that viral escape from TLR7/8 pressure is not easy. Induction of the innate immune response can also be initiated through detection of viral RNA by retinoic acid-inducible gene I (RIG-I)-like receptors (RLRs). RIG-I is an RNA helicase that is expressed in epithelial and fibroblastic cells as well as dendritic cells and macrophages [100]. Although RIG-I mainly senses uncapped viral ssRNAs, it also recognizes the dimeric capped retroviral HIV-1 RNA found in mature virions [100]. Monomeric HIV-1 RNA was an even better inducer of RIG-I than dimeric HIV-1 RNA [100]. Secondary structures in the viral ssRNA such as the TAR hairpin are better inducers of cytokine expression than HIV-1 RNA oligos without (predicted) extensive secondary structure [101]. However, RIG-I signaling is efficiently inhibited by the HIV-1 protease that depletes RIG-I from the cytoplasm [100].

Another interferon-induced antiviral factor is RNase L (also named 2',5'-oligoadenylate-dependent RNase L or 2-5A-dependent ribonuclease), an enzyme that cleaves viral RNA predominantly at UpA and UpU dinucleotides [102,103]. The enzyme 2',5'-oligoadenylate-synthetase (OAS) that is needed to activate RNase L is itself induced by the HIV-1 TAR RNA hairpin [104,105], a regulatory structure present at the 5’-end of all viral transcripts. However, OAS binding can be inhibited *in vitro*, and most likely also *in vivo*, by addition of the HIV-1 Tat protein, which competes efficiently for TAR binding [104]. RNase L expression decreases HIV-1 replication when the human RNase L gene is artificially introduced into the viral genome [106], but RNase L is not effective during natural HIV-1 infection [107]. After HIV-1 infection, the RNase L pathway is downregulated through induction of RNAse L inhibitor (RLI) expression [108]. UpA and UpU dinucleotide frequencies, the main targets for RNase L cleavage, are not particularly reduced in HIV-1 genomes [28], in line with the lack of RNAse L pressure on HIV-1 RNA.

SAMHD1 (sterile alpha factor and HD-domain 1) is another HIV-1 restriction factor that was recently identified [109,110]. SAMHD1 is a deoxynucleoside triphosphate triphosphohydrolase [111] that can be upregulated by type I and II interferons and by TLR ligands. It regulates dNTP pools in non-cycling myeloid cells such as macrophages, and possibly also in quiescent CD4+ T cells (for a review, see [112]). SAMHD1 is counteracted by the retroviral Vpx protein that is encoded by SIV and HIV-2, but this gene is lacking from the HIV-1 and FIV genomes [109,110]. Despite the lack of Vpx function, HIV-1 is able to replicate in non-cycling myeloid cells, albeit a low levels, possibly because HIV-1 RT has a very high affinity for dNTPs [112]. SAMHD1 efficiently hydrolyses all four dNTPs, although it needs dGTP to initiate hydrolysis, suggesting that dGTP might be the preferred substrate for the enzyme [112]. As SAMHD1 affects the total dNTP pool, it remains uncertain if SAMHD1 can influence HIV-1 genome composition through induction of nucleotide pool imbalances, but the subject warrants further study.

The human schlafen 11 (SLFN11) protein that is induced by pathogens via the interferon regulatory factor 3 (IRF3) pathway inhibits HIV protein synthesis in a codon-usage based manner. SLFN11 binds to tRNAs and counteracts the changes in the cellular tRNA pool observed after HIV infection [113]. The rare tRNAs that are increased by HIV-1 are repressed in the presence of SLFN11 [113]. However, the HIV-1 encoded Vpu protein effectively depletes IRF3 during HIV infection [114], thus antagonizing the induction of SLFN11. This suggests that SLFN11 does probably not have an appreciable effect upon HIV-1 replication and codon usage *in vivo*, as there is little need for the virus to escape from this putative restriction factor.

One may cautiously conclude that antiviral factors of the innate immune system are unlikely to influence the nucleotide composition of HIV-1 in an appreciable way, neither by directly mutating the genome nor through HIV-1 escape from the antiviral pressure imposed by these factors.

### Nucleotide bias problem

A biased nucleotide composition will influence the phylogenetic analysis of viral genome sequences. The same holds for amino acid sequences if the typical nucleotide bias results in a biased protein composition, as is the case for HIV. All phylogenetic methods used today are sensitive to nucleotide composition in a sequence alignment. Sequences with similar base frequencies will be clustered together in the resulting tree, whether or not this results from shared ancestry [3]. In practice, this limitation will not affect most studies that involve intraspecies or intragenus comparisons, in which the viral genomes do possess a similar base composition that is inherited from a common ancestor. Tree analyses involving different retrovirus families could however lead to erroneous conclusions about clade relationships and divergence times [3,5,6]. And even relationships between distinct virus families can be obscured by genomic nucleotide composition. For instance the A-rich influenza virus of the (−) ssRNA family of Orthomyxoviridae has a codon use comparable to HIV-1, in contrast to the C-rich retrovirus murine leukaemia virus (MLV) that consequently appears to be more distinct from HIV-1 than influenza virus [47]. Manipulating sequence alignments, e.g. leaving out the third, most variable codon position does not appreciably improve the accuracy of the resulting phylogeny, as all three codon positions are affected by nucleotide bias, with the exception of U at the second codon position [3]. However, uridines at second codon positions are generally quite conserved among retroviruses, and do thus not provide a solution to resolving phylogenetic relationships [3].

### Nucleotide composition of retroviral versus host DNA

We have focused thus far on the HIV-1 RNA genome. Upon infection of the host cell, the HIV-1 genomic RNA is converted into dsDNA by reverse transcription. The newly made HIV DNA, complexed with viral and cellular proteins, is actively imported into the nucleus through the nuclear pore, such that HIV is not dependent on cell division for nuclear import and subsequent DNA integration into the host genome. Can the biased nucleotide composition also affect the function of the HIV-1 DNA genome?

HIV-1 integration sites can be found on all chromosomes, but integration is not completely random. Viral pre-integration complexes (PICs) target specific chromosomal locations that are associated both with gene density and transcriptional activity (“integration hotspots”) [115]. What would be the effect when an AT-rich DNA such as the HIV genome integrates into a genomic location that is not AT-rich? Although the human reference genome is AT-rich (60%) and GC-poor (40%), much like HIV-1 genomes (strain HXB2: 57% AT, 43% GC), there is considerable variation in local sequence composition. The human genome consists of mosaics of isochores, which are megabase-sized DNA stretches with a homogeneous base composition [116]. In addition, CpG islands, short sequences of 0.5-2 kb in size that are rich in GC but low in methylated CpG dinucleotides, are distributed throughout the human genome (see [115]). CpG islands are associated with gene-rich regions, and their frequency is positively associated with the GC-richness of the isochores [117].

Retroviruses have been shown to favour isopycnic and compartmentalized integration, e.g. genomic integrations were found in chromosomal locations with a base composition similar to that of the viral genome [49,118,119]. Why would they favour isopycnic integration? Are GC-rich sequences somehow removed from AT-rich genomic regions? An answer to this question may come from studies involving the integration preference from other retroelements, such as Alu repeats. Alu repeats are retroelements that are classified as SINEs (Short Interspersed Elements) due to their short length (~300 bp) (for a review, see [120]). Because Alu has no coding capacity, it depends on other retroelements for retrotransposition. Only a minority of the 1 million Alu copies in the human genome are retrotranspositionally active at present. The GC-rich Alu repeat sequences do target AT-rich isochores; there is a relatively high density of younger integrations in those locations, but they are probably unstable there [121]. As a result, Alu integrations (especially the older ones) are mainly found in a GC-rich chromosomal environment [121]. Retroviral DNA genomes, whether GC-rich or GC-poor, commonly target the open chromatin of GC-rich isochores, but proviral integrations from GC-poor viruses are probably unstable in a GC-rich environment, similar to GC-rich proviruses being comparatively unstable in a GC-poor location [121]. A reconstructed infectious clone of the AT-rich human endogenous retrovirus-K (HERV-K) HML-2 provirus (containing approximately 60% AT) integrated into GC-rich regions far more often than would be expected based on the ancient HERV-K provirus locations [122]. This may suggest that HERV-K integrations may be deleted from GC-rich chromosomal fragments over time, although no such activity is currently known.

Comparing HIV-1 integration sites in the human genome in both primary cells and cell lines, Mitchell *et al*. showed that HIV-1 may prefer GC-rich isochores for integration, but it disfavours the regions around CpG islands, in contrast to the GC-rich (64%) MLV [115]. HIV-1 favours active genes for integration, but does not integrate into the gene subset with the highest expression level [115]. Integration is thought to involve interaction of the PIC with proteins bound to the host DNA [115]. Possibly, HIV-1 PICs cannot interact with the specific (regulatory) proteins bound to chromosomal DNA in or in close proximity to CpG islands, in contrast to MLV PICs, but instead bind transcription factors present in active transcription units.

In summary, HIV-1 prefers, like other retroviruses and repeat elements such as Alu repeats, the open, active chromatin in GC-rich isochores for DNA integration, but being GC-poor the provirus may be somewhat unstable at that location. This may not be a problem for HIV-1, as integration into cells of the germ-line has not been described until now, and proviral integrations in relatively short lived cells will probably be stable for the duration of the cell’s life. Studies examining the stability of HIV-1 integrations in long-term infected cells have not been performed yet. However, as ancient endogenous lentivirus genomes with relatively low GC-counts have been detected in several mammalian species, and HERV-K integrations survive in the germ-line while being GC-poor (the 34 HERV-K proviruses analysed have 35-38% GC) [37], it is likely that at least some retroviral GC-poor integrations can survive for a long time. Furthermore, GC-rich isochores are decreasing in most mammalian orders including primates, murids and carnivores when compared to other mammalian orders such as lagomorphs, perissodactyls and cetartiodactyls, probably due to the higher recombination rate in GC-rich genomic regions in the former mammalian lineages [123]. It has been estimated that the isochore structure is disappearing entirely from the human and chimpanzee genome, but not yet from the baboon genome [124]. Human and chimpanzee genomes are homogenising to an average GC-content of 42% [124], similar to HIV DNA (43% GC) and SIV DNA from chimpanzees (42% GC). Possibly, somewhere in the future, HIV proviruses will be more stable in a homogenous host genome where the rate of homologous recombination has decreased, which is important should HIV-1 ever infect the germ-line.

## Conclusions

The HIV-1 RNA genome is particularly rich in A-nucleotides while the C-content is low. HIV-1 is one of the most variable viruses known, yet it is able to maintain this highly biased nucleotide composition. Comparing HIV-1 genomes from the beginning of the epidemic with more recent isolates shows that the nucleotide composition is extremely stable over the past 30 years. Even the cross-species transmission events from chimpanzees to humans did not substantially change the nucleotide composition of HIV-1 genomes of group M, N and O viruses, although the single HIV-1 group P virus seem to contain slightly less adenine and somewhat more C nucleotides, in line with non-chimpanzee primate SIVs and HIV-2, and its supposed descent from SIVgor. The base composition of HIV-1 has been linked to differences in pathogenicity of the subtypes, whereby a base composition that deviates most from that of the human host correlates with increased virulence [81]. In that context, the slightly different base composition of HIV-1 compared to HIV-2 may also correlate with the increased pathogenicity of the former.

The A-richness of the HIV-1 genome may have been caused by a distinct mutation pattern of the viral RT polymerase, but there could also have been evolutionary pressure to select an A-rich RNA genome. Further research is needed to identify possible RNA functions imposed by the A-abundance. No evidence has been reported that factors of the innate immune system shape the nucleotide composition of the viral genome, either by direct mutational activity or indirectly through viral escape.

## Competing interests

The authors declare no competing interests.

## Authors' contributions

ACvdK conceived the review topic and ACvdK and BB drafted the manuscript. Both authors read and approved the final manuscript.
